# Influence of the Blends of Sheep's and Goat's Milk on the Functional Properties and Volatile Compounds of Lighvan Cheese During Ripening: A Comparative Study

**DOI:** 10.1002/fsn3.4543

**Published:** 2024-10-23

**Authors:** Solmaz Saremnezhad, Mostafa Soltani, Ali Tekin, Hilal Kanmaz, Didem Sahingil, Büşra Kaya, Yasemin Gökçe, Ali Adnan Hayaloglu

**Affiliations:** ^1^ Department of Food Science and Technology, Faculty of Pharmacy Islamic Azad University of Medical Sciences Tehran Iran; ^2^ Nutrition and Food Sciences Research Center, Tehran Medical Sciences Islamic Azad University Tehran Iran; ^3^ Department of Food Technology Keban Vocational School Elazığ Türkiye; ^4^ Department of Food Engineering Inonu University Malatya Türkiye

**Keywords:** bioactive, goat's milk, Lighvan, sheep's milk, volatile

## Abstract

In this study, the influence of different ratios of sheep's to goat's milk (100:0, 75:25, 50:50, 25:75, and 0:100) on the content of functional metabolites and volatiles in Lighvan cheese throughout ripening was investigated. The samples were ripened under brine for 90 days and assessed in terms of variations in chemical composition, proteolysis, angiotensin‐converting enzyme, α‐amylase, and α‐glucosidase inhibitory activities, antioxidant power, total free amino acids, gamma amino butyric acid, free fatty acids, and volatile compounds. Results showed that by increasing the proportion of goat's milk, the samples exhibited higher antioxidant effect and inhibition of the angiotensin‐converting enzyme during ripening. After 90 days, an increase in α‐glucosidase and a slight decrease in α‐amylase inhibitory activities were observed in Lighvan cheese samples that were produced with 50%, 75% and 100% of goat's milk. In contrast, higher contents of sheep's milk led to a significant increase in the concentration of esters, terpenes and alcohols and a slight increase in gamma‐aminobutyric acid. The highest concentration of saturated and unsaturated fatty acids was observed in the cheese prepared with 100% raw sheep's milk after 90 days of ripening. The results revealed the possibility of making a functional white‐brined cheese by monitoring the type and ratio of the used milk and ripening time.

## Graphical abstract

By changing the ratio of sheep's to goat's milk and controlling the ripening time in brine, it is possible to improve the production of functional metabolites with bioactive properties in Lighvan cheese and in addition to benefiting from the nutritional value of this cheese, control some diseases such as diabetes, blood pressure, etc.

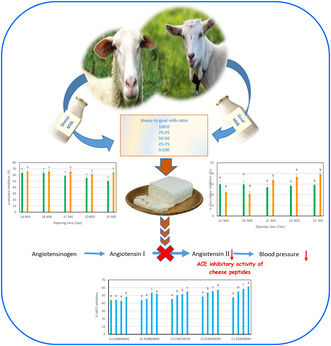

## 1 Introduction

Nowadays, production of functional foods has increased due to their health benefits. Proteins and fats are important sources of bioactive compounds that can be hydrolyzed into peptides, amino acids and various fatty acids with biological activities. Milk is rich in proteins and fats and is a suitable potential source of bioactive compounds. Enzymatic hydrolysis or fermentation of milk with proteolytic microorganisms, e.g., lactic acid bacteria (LAB) can cause the formation of bioactive peptides such as those with antihypertensive, antimicrobial, antioxidant, immunoregulatory, or mineral‐binding effects (Kocak et al. 2020; Campagnollo et al. 2022).

Cheeses are a large group of fermented milk‐based foods, produced in various types and flavors. Texture quality (hardness or softness) is the most common criterion for the classification of cheeses (Fox and McSweeney 2017). Lighvan is a type of starter‐free white cheese with a semihard texture, which mainly is produced in traditional form in western regions of Iran (East‐Azerbaijan province). It is usually made from raw sheep's milk but sometimes traditional producers may also add small quantities of goat's milk to the sheep's milk (Hayaloglu 2017). Cheese production begins with filtration, cooling the raw milk (22°C–23°C) and mixing with lamb rennet. After 2 h, the coagulum is drained and cut into small pieces (2 × 2 cm). Weights are used to facilitate draining of the whey from the small coagulum pieces. The resultant curd is cut into 15 × 15 cm cubes. The cubes are transformed to a basin and covered with dry salt so that the remaining whey can drain off. Finally, the curds are put in 10%–12% brine in tin containers and stored in cold rooms for at least 90 days to mature.

During ripening, various physico‐chemical changes occur in the cheese, such as glycolysis, lipolysis, and proteolysis. The different chemical composition of goat's and sheep's milk (e.g., the average content of protein, lactose, fat and non‐fat solids) can affect the characteristics of the cheese prepared from the mixed milk and lead to the formation of different compounds and metabolites.

To date, many studies have been conducted on various qualitative aspects of Lighvan cheese, but there is not much information available on the effects of milk types and portions on the formation and content of functional compounds during the ripening of this type of cheese. Considering the role of α‐amylase and α‐glucosidase in the hydrolysis of carbohydrates, inhibiting the activity of these enzymes could be an effective approach in controlling blood sugar, especially in diabetic patients. On the other hand, hypertension is a common cardiovascular disease that originates from sedentary lifestyle and unhealthy diet. ACE as a significant enzyme in rennin‐angiotensin system, is responsible for converting “angiotensin I” into an octapeptide “angiotensin II,” which causes blood pressure to rise. Food‐origin peptides with ACE inhibitory activity are considered as a safe, stable, and hypoallergenic alternatives or adjuvants for treatment of hypertensive disorders (Wang et al. 2024). Lighvan cheese as a fermented dairy product could be a good source of bioactive peptides. In a relatively new study, Saidi et al. (2020) profiled functional neurotransmitters of Lighvan cheese made from raw sheep's milk and reported the elevation of tyramine, gamma‐aminobutyric acid (GABA), dopamine, and glutamate, during the ripening. Against this background and the important role of aromatic compounds in Lighvan cheese flavor, the current research aimed to determine the effects of sheep and goat's milk proportions on the functional properties and volatile compounds of this type of cheese during its ripening.

## 2 Materials and Methods

Raw sheep's milk (total solids 16.59%, fat 6.64%, protein 5.84% and pH 6.67) and goat's milk (total solids 13.33%, fat 3.97%, protein 3.44% and pH 6.74) collected from a local indigenous sheep and goat population in the Kordan region (Alborz province, Iran). Standard cheese‐making calf rennet (2235 international milk coagulation units/g, Chr‐Hansen Horsholm, Denmark) was used as coagulant. All other chemicals were of analytical grade and supplied from Merck Co. Inc. (Darmstadt, Germany).

### 2.1 Production of Lighvan Cheese

Three separate cheese making trials were performed in a consecutive week. The blend of sheep and goat's milk were used to produce Lighvan cheese. The mixing ratio was as follows:

A: 100% Sheep's milk + 0% Goat's milk.

B: 75% Sheep's milk + 25% Goat's milk.

C: 50% Sheep's milk + 50% Goat's milk.

D: 25% Sheep's milk + 75% Goat's milk.

E: 0% Sheep's milk + 100% Goat's milk.

The milk mixture was heated to 30°C. Standard cheese‐making rennet was used to clot the milk. After 2 h, the coagulum was cut and the whey inside the coagulum was drained by placing it in a cotton bag and putting hangs on the bag. After about 2 h, the curds were cut into cubes and placed in 16% w/w NaCl solution for 6 h at 20°C. Ripening was carried out in 12% w/w salt solution at a temperature of 8 ± 1°C for 90 days. The Lighvan cheese production was performed in three replicates and cheeses were sampled after 1, 30, 60, and 90 d of ripening. All analyses were performed in triplicate, except where stated in the text.

### 2.2 Gross Chemical Analysis

Total solids, fat, pH, and total nitrogen contents of cheese samples were determined according to the methods described in Soltani, Guzeler, and Hayaloglu (2015).

### 2.3 Proteolysis

The water‐soluble and 12% trichloroacetic acid (TCA)‐soluble fractions were prepared according to the procedure of Hayaloglu et al. (2005). The nitrogen content of each fraction was determined by Kjeldahl method and reported as a percentage of the total nitrogen in the cheese. The degradation profile of the casein fractions during ripening was monitored by urea‐poly acrylamide gel electrophoresis (urea‐PAGE) and the gels were stained and de‐stained by the method given its detail in Soltani, Guzeler, and Hayaloglu (2015).

The concentrations of individual and total free amino acids (TFAA) were measured on water‐soluble fraction (WSF) of samples as described by Uruc et al. (2022).

### 2.4 Angiotensin‐Converting Enzyme‐Inhibitory (ACE‐i) Activity

The ACE‐i activity of the cheese samples was measured by the RP‐HPLC as given in detail by Sahingil et al. (2019).

### 2.5 Antioxidant Activity

ABTS (2,2′‐azino‐bis (3‐ethylbenzothiazoline‐6‐sulfonic acid)) radical scavenging activity of WSF of the samples was measured by the method described by İncili et al. (2023).

### 2.6 α‐Amylase and α‐Glucosidase Inhibitory Activity

The α‐glucosidase and α‐amylase inhibition power were measured according to the method of Ayyash et al. (2018) and Cam et al. (2020) with some slight modifications.

For α‐amylase inhibition, α‐amylase (100 μL, 1.0 unit/mL) was added to WSF (100 μL) and incubated in a water bath (37°C) for 5 min. Then, starch (250 μL, 1% w/v) was used to initiate the reaction. DNS reagent (200 μL, 1% 3,5‐dinitrosalicylic acid, and 12% sodium potassium tartrate) was added and the mixture kept at 100°C for 15 min. After cooling and addition of distilled water (2 mL), the absorbance (Abs) of the mixture was read at 540 nm. Equation (1) was used to calculate the α‐amylase inhibition percentage.

$$\text{Inhibition}\%=\left[1-\frac{\mathrm{Abs}\;\text{sample}-\mathrm{Abs}\;\text{blank}}{\mathrm{Abs}\;\text{control}}\;\right]$$

For α‐glucosidase inhibition, α‐glucosidase (100 μL, 1.0 unit/mL prepared in 0.1 M potassium phosphate buffer at pH 6.8) was mixed with diluted sample (50 μL). The mixture was incubated at 37°C for 10 min. *p*‐Nitrophenyl α‐d‐glucopyranoside (50 μL, 5 mM) was added to the tubes. The resultant reaction was stopped by Na_2_CO_3_ (2.5 mL, 0.1 M) after incubation at 37°C for 30 min. The absorbance was determined at 400 nm and the inhibition rate was calculated by equation (1).

### 2.7 Free Fatty Acid (FFA) and Volatile Analysis

Two grams of grated cheese was mixed with anhydrous Na_2_SO_4_ (2.5 g) and Heptanoic acid added as the internal standard. The mixture was vortexed for 1 min and centrifuged (Hettich Universal 32R, Tuttlingen, Germany) at 1380 *g*, 0°C for 10 min (Tekin and Güler 2019). Gas chromatography (GC) was used for the analysis of samples. GC conditions were the same as reported by Tekin and Güler (2019) and the results were expressed in terms of mg kg^−1^. Volatile compounds were analyzed according to the method described by Sulejmani, Sahingil, and Hayaloglu (2020).

### 2.8 Statistical Analyses

The data are reported as mean ± SD of at least three replicates and analyzed statistically using one‐way ANOVA. The data were compared by Duncan's multiple comparison test (*α* = 0.05). Statistical analyses were carried out using SPSS software (IBM Corp. 2013).

## 3 Results and Discussion

### 3.1 Cheese Gross Composition

The changes in the gross composition of the Lighvan cheese samples during ripening are shown in Table 1. The proportion of goat's and sheep's milk had a significant influence on pH, dry matter, fat‐in‐dry matter (FDM), and protein contents (*p* < 0.05). An increase in the proportion of sheep's milk and progress of ripening caused a significant decrease (*p* < 0.05) in pH in each treatment. The activity of the natural microbiota of the milk, the fermentation of lactose and formation of lactic acid, release of FAAs and FFAs through proteolysis and lipolysis could be the reasons of the pH drop during ripening (Soltani, Guzeler, and Hayaloglu 2015). The lower pH of cheeses made from higher proportions of sheep's milk is related to the lower pH and higher dry matter and lactose contents of sheep's milk compared to goat's (Miloradovic et al. 2017). Sample A had the highest dry matter among the samples. Significant elevation of dry matter in cheeses made from higher concentrations of sheep's milk is due to the higher dry matter and protein of sheep's milk compared to goat's milk. The curd particles of milk with high protein content have high water holding capacity, so they form large volumes of coarse protein fractions with high whey drainage which results in production of a cheese with low moisture content (Soodam and Guinee 2018). Ripening of samples for up to 60 days led to an increase in dry matter, probably due to the exchange of moisture inside the cheese with the salt ions in the brine to restore the osmotic pressure equilibrium between the cheese and the brine (Soltani, Guzeler, and Hayaloglu 2015). The decrease in dry matter after 60 days could be the result of an increase in the concentration of water‐soluble compounds due to proteolysis, and lipolysis of the samples and their release into the brine medium (Soltani et al. 2022).

**TABLE 1:** Chemical composition and ACE‐inhibitory activity of Lighvan cheeses manufactured with different ratios of sheep to goat's milk, during ripening.

	Day	A	B	C	D	E
pH	1	4.65 ± 0.02^aB^	4.73 ± 0.01^bB^	4.77 ± 0.01^bB^	4.78 ± 0.07^bB^	5.28 ± 0.07^cC^
	30	4.71 ± 0.03^aC^	5.12 ± 0.01^bC^	5.14 ± 0.01^bC^	5.17 ± 0.06^bC^	5.42 ± 0.04^cD^
	60	4.39 ± 0.04^aA^	4.56 ± 0.01^bA^	4.66 ± 0.06^cA^	4.72 ± 0.06^dB^	5.12 ± 0.02^eB^
	90	4.34 ± 0.06^aA^	4.53 ± 0.09^bA^	4.60 ± 0.05^cA^	4.64 ± 0.10^cA^	4.88 ± 0.10^dA^
Dry matter (%)	1	47.77 ± 0.48^eA^	46.02 ± 0.52^dA^	45.74 ± 0.42^cA^	40.89 ± 0.51^bA^	39.88 ± 0.78^aA^
	30	48.08 ± 0.99^eB^	46.15 ± 0.25^dA^	45.79 ± 0.63^cA^	41.64 ± 0.48^bB^	40.01 ± 0.77^aA^
	60	50.95 ± 0.81^eD^	48.14 ± 0.93^dB^	47.34 ± 0.36^cB^	42.18 ± 0.63^bC^	40.62 ± 0.50^aA^
	90	49.67 ± 0.61^eC^	47.79 ± 0.7^dB^	45.94 ± 0.75^cA^	42.1 ± 0.85^bC^	40.18 ± 0.64^aA^
Fat in dry matter (%)	1	56.14 ± 0.19^eC^	55.82 ± 0.32^dD^	53.27 ± 0.11^cC^	50.93 ± 0.24^bD^	47.38 ± 0.28^aC^
	30	55.91 ± 0.21^eB^	54.33 ± 0.18^dB^	52.54 ± 0.34^cB^	50.05 ± 0.25^bB^	46.84 ± 0.15^aB^
	60	55.36 ± 0.11^eA^	53.89 ± 0.23^dA^	51.89 ± 0.18^cA^	49.44 ± 0.15^bA^	46.30 ± 0.22^aA^
	90	56.02 ± 0.33^eBC^	54.75 ± 0.17^dC^	53.17 ± 0.23^cC^	50.36 ± 0.20^bC^	47.13 ± 0.19^aC^
Protein (%)	1	17.49 ± 0.49^dA^	17.06 ± 0.0^cA^	16.22 ± 0.16^bA^	15.46 ± 0.77^aA^	15.34 ± 1.86^aA^
	30	17.37 ± 3.01^dA^	16.69 ± 0.38^cA^	16.13 ± 0.14^bA^	15.28 ± 0.24^aA^	15.29 ± 0.55^aA^
	60	17.58 ± 0.00^eA^	17.09 ± 0.38^dA^	16.25 ± 0.62^cA^	15.84 ± 0.45^bB^	15.59 ± 0.04^aB^
	90	19.89 ± 0.01^eB^	18.18 ± 0.00^dB^	17.73 ± 0.29^cB^	17.18 ± 0.95^bC^	16.29 ± 0.09^aC^
GABA (mg/100 g cheese)	1	3.42 ± 0.01^cB^	2.68 ± 0.08^cA^	2.36 ± 0.05^bA^	1.50 ± 0.02^aA^	2.30 ± 0.12^bA^
	30	3.17 ± 0.01^bB^	2.59 ± 0.23^bA^	2.16 ± 0.02^aA^	2.05 ± 0.11^aB^	2.87 ± 0.17^bB^
	60	3.12 ± 0.09^bB^	2.66 ± 0.19^bA^	2.36 ± 0.06^abA^	2.06 ± 0.09^aB^	3.05 ± 0.03^bB^
	90	2.61 ± 0.11^bB^	2.69 ± 0.1^bA^	2.61 ± 0.03^bA^	2.01 ± 0.23^aB^	3.06 ± 0.01^bB^
ACE‐inhibitory activity (%)	1	50.86 ± 0.72^aA^	55.51 ± 0.27^bA^	82.91 ± 1.14^cA^	84.68 ± 1.09^dA^	90.06 ± 0.72^eB^
	30	62.59 ± 0.25^bB^	59.03 ± 0.01^aB^	84.49 ± 0.40^cB^	90.62 ± 0.45^dC^	90.82 ± 1.05^dC^
	60	68.78 ± 0.28^bC^	67.63 ± 0.38^aC^	86.37 ± 0.83^cC^	91.21 ± 0.82^dC^	91.25 ± 0.90^dC^
	90	71.34 ± 0.55^aD^	72.00 ± 0.61^bD^	89.36 ± 1.334^cD^	89.36 ± 0.43^cB^	89.02 ± 0.01^cA^
TFAA (mg/ 100 g cheese)	1	410.44 ± 0.56^cA^	322.65 ± 0.66^bA^	268.06 ± 0.91^bA^	235.72 ± 0.55^aA^	199.66 ± 0.3^aA^
	30	425.18 ± 0.30^dA^	346.27 ± 0.38^cA^	305.89 ± 0.62^bB^	241.93 ± 0.34^aA^	274.28 ± 0.65^aB^
	60	502.09 ± 0.56^dB^	443.5 ± 0.46^cB^	379.1 ± 0.66^bC^	370.18 ± 0.77^bB^	320.97 ± 0.76^aC^
	90	549.04 ± 1.66^dC^	472.14 ± 0.56^cC^	422.2 ± 0.34^bD^	400.85 ± 0.45^bC^	361.22 ± 0.40^aD^
The difference between free hydrophobic amino acids on days 90 and 1		87.05	84.5	115.43	117.65	123.47

*Note:* Different upper‐case superscripts show statistical differences (*p* < 0.05) in a sample at different ripening times for each of the parameters measured. Different lower‐case superscripts show statistical differences (*p* < 0.05) between cheese samples at a constant ripening time for each of the parameters measured. A: 100% Sheep's milk + 0% Goat's milk, B: 75% Sheep's milk + 25% Goat's milk, C: 50% Sheep's milk + 50% Goat's milk, D: 25% Sheep's milk + 75% Goat's milk, E: 0% Sheep's milk + 100% Goat's milk.

Abbreviations: ACE, angiotensin‐converting enzyme; GABA, gamma‐amino‐butyric acid; TFAA, total free amino acids.

Changes in the dry matter content of samples also led to significant changes in their FDM during ripening (*p* < 0.05). The samples with a higher ratio of sheep's milk (samples A and B) had the highest FDM values during ripening (*p* < 0.05). The protein content is an important component of white cheese. The type of milk and the ripening time had significant effects on the protein content of the cheese samples (*p* < 0.05). The lower protein content of goat's milk compared to sheep's milk led to lower protein levels in the samples with higher proportions of goat's milk (samples E and D).

### 3.2 Proteolysis Assessment

**FIGURE 1:**
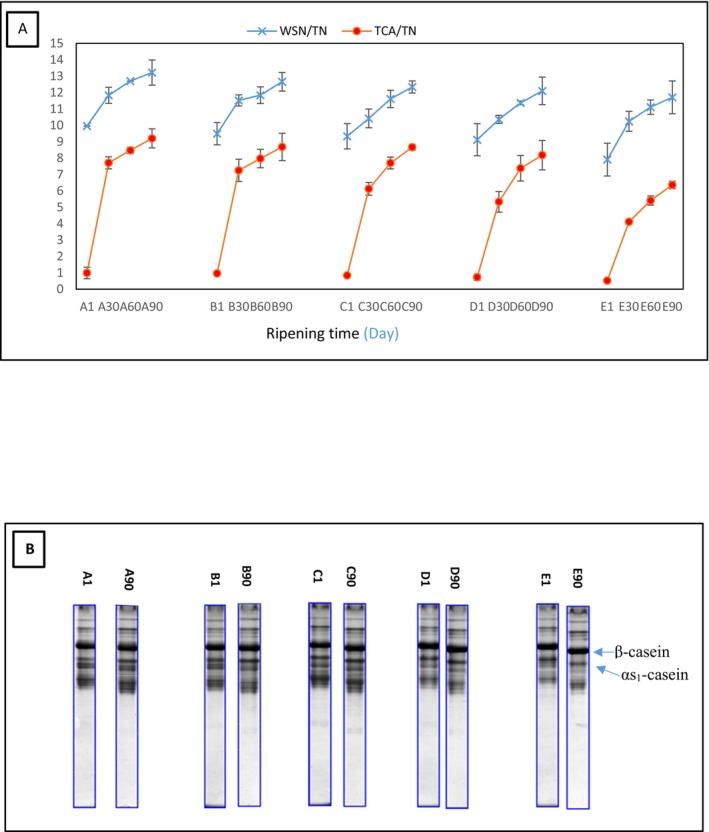
(A) The trend of WSN/TN and TCA/TN changes and (B) the electrophoretograms of Lighvan cheeses manufactured with different ratios of sheep and goat's milk during the ripening 569 period. A: 100% Sheep's milk + 0% Goat's milk, B: 75% Sheep's milk + 25% Goat's milk, C: 50% Sheep's milk + 50% Goat's milk, D: 25% Sheep's milk +75% Goat's milk, E: 0% Sheep's milk + 100% Goat's milk TCA, tri‐chloro acetic acid soluble fractions; TN, total nitrogen; WSN, water soluble nitrogen.

Figure 1A shows the trend of water‐soluble nitrogen (WSN)/TN and TCA/TN changes in different Lighvan samples during the ripening period. WSN/TN and TCA/TN as good indices of proteolysis increased with increase of ripening time in all samples. An increase in the content of sheep's milk led to an increase in primary and secondary proteolysis. The electrophoretograms (Figure 1B) of the samples confirm these results. The intensity of proteolysis was the lowest in sample E due to the lower amount of protein, casein, and lower number of proteolytic enzymes in goat's milk compared to sheep's milk (Park et al. 2007).

Proteolysis, the breakdown of caseins by the activity of nonstarter lactic acid bacteria (NSLAB), microbial enzymes, and rennet lead to the release of FAAs during the ripening of a cheese (Soltani et al. 2022). The TFAA content (Table 1) showed an approximately increasing trend as ripening progressed. A decrease in TFAA content was observed with the decrease in sheep's milk proportion, so that sample E had the lowest amount of TFAA during ripening. The higher degree of proteolysis in the samples with higher concentrations of sheep's milk can be seen in Figure 1. As can be seen in this figure, the WSN content increased in all cheese samples as ripening progressed. The measurement of TCA‐SN concentration as a good index for the study of secondary proteolysis of cheese, also showed an increasing trend with progress of ripening and increase in the ratio of sheep to goat's milk. The highest and lowest levels of WSN/TN and TCA/TN were observed on the 90thday of ripening in samples A and E, respectively, which is consistent with the results of the TFAA content of the samples.

GABA has several physiological functions such as regulation of blood pressure, antidepressant, and antidiabetic properties, stimulation of immune cells, and regulation of the sleep–wake cycle (Saremnezhad et al. 2021). In the current study, slight changes in GABA content were observed with the progress of ripening (Table 1). No significant changes in GABA were observed in samples prepared with 100%, 75%, and 50% sheep's milk during ripening (*p* > 0.05). When the proportion of goat's milk was increased to over 50% (samples D and E), a slight increase in GABA content was observed up to day 30 of ripening (*p* < 0.05), but the changes from day 30 to 90 were not significant (*p* > 0.05). Glutamic acid is the precursor of GABA biosynthesis. Decarboxylation of glutamate by glutamate decarboxylase (EC 4.1.1.15) from starters and NSLAB in milk is known to be the mechanism for GABA biosynthesis within the cheese matrix. On the other hand, glutamine is also synthesized from glutamate by glutamine synthase (EC 6.3.2.1) and competes with GABA for glutamate (Redruello et al. 2020). The natural microbiota of milk has a key role in the synthesis of GABA, especially in raw milk cheeses. The duration of ripening, the type of milk, thermal treatment, the pH of the medium, and the microbial flora (Dhakal, Bajpai, and Baek 2012) are important factors that influence the GABA content of cheeses. The optimal pH for maximum GABA production is species‐dependent. Different LAB, produce GABA in different pH ranges (4.5–8; Dhakal, Bajpai, and Baek 2012). In the present study, the pH of the cheese samples on the 90th day of ripening ranged from 4.34 to 4.88 (Table 1). It seems that the type of milk (sheep, goat, or a mixture of both in terms of protein and amino acid composition), the type of microflora, and the pH of the cheese play key roles in obtaining these results. Further studies are needed to investigate the relationship between milk type, microflora, and pH with GABA synthesis in Lighvan cheese.

### 3.3 ACE Inhibitory Activity

In general, with increase in the proportion of goat's milk, the ACE inhibitory activity of cheeses increased. Sample E possessed the highest ACE inhibitory activity. A slight decrease in ACE inhibition power of cheeses prepared with 75%, and 100% goat's milk (samples D and E) was observed after 60 days of ripening (Table 1). Various studies have found an increase in ACE inhibitory activity in different cheeses up to a certain level of proteolysis (Hossain et al. 2020; Kocak et al. 2020; Ayyash et al. 2021; Ramos et al. 2022). The natural formation of peptides in a cheese depends on the proteolytic power of fermenting microorganisms during ripening. The peptides could break down into fragments with lower molecular weight and weaker ACE inhibitory effect during ripening (Kocak et al. 2020). The high inhibitory effect of the Lighvan cheese made from 100% raw goat's milk during the ripening could be attributed to the amino acid sequence of the peptide fragments from goat's milk and the proteolytic power of the native microbiota of this type of milk (da Silva et al. 2019).

### 3.4 Antioxidant Activity

**FIGURE 2:**
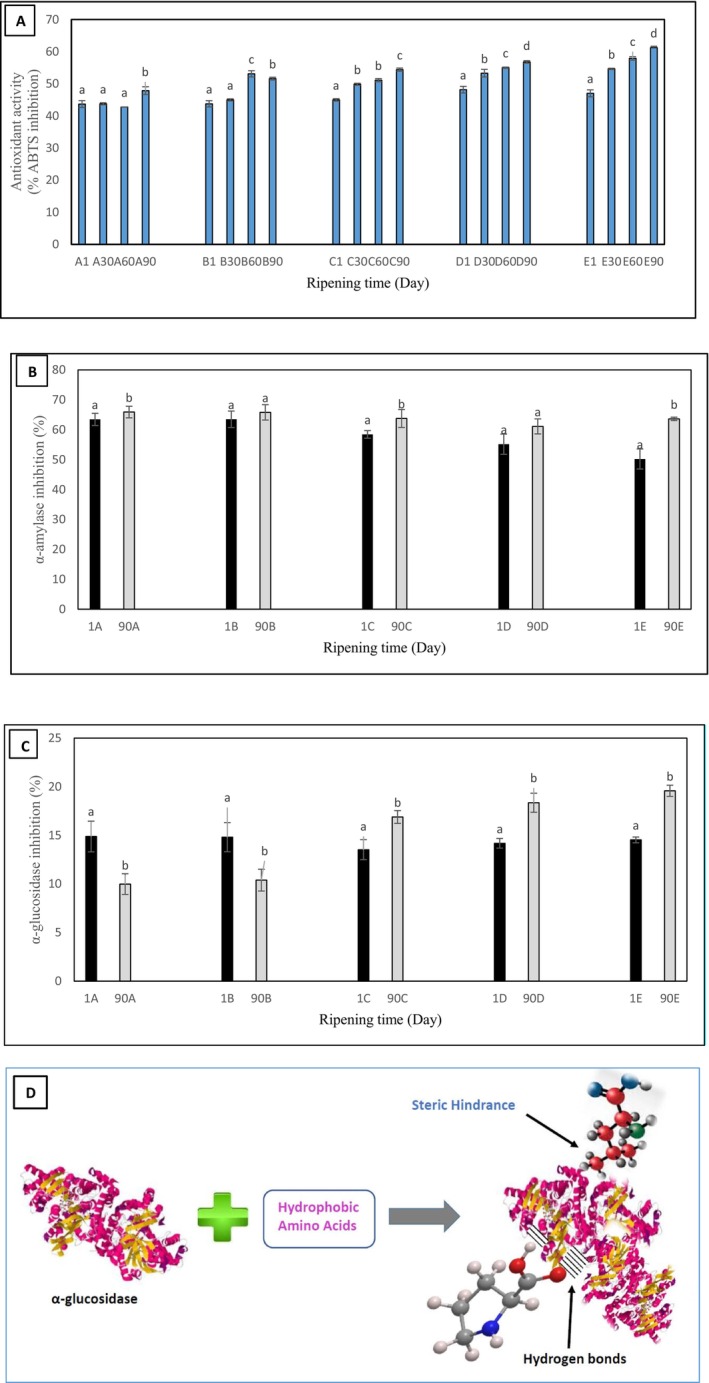
(A) The antioxidant activity, (B) α‐amylase inhibitory activity, (C) α‐glucosidase inhibitory activity of Lighvan cheeses manufactured with different ratios of sheep and goat's milk during the ripening period. (D) The mechanism of action of hydrophobic amino acids on the inhibition of α‐576 glucosidase activity. Different letters above each set of data indicate the significant difference (*p* < 0.05). A: 100% Sheep's milk + 0% Goat's milk, B: 75% Sheep's milk + 25% Goat's milk, C: 50% Sheep's milk + 50% Goat's milk, D: 25% Sheep's milk + 75% Goat's milk, E: 0% Sheep's milk + 100% Goat's milk.

Antioxidant bioactive peptides can be formed during the proteolysis of a cheese (Hossain, Khetra, Ganguly, Kumar, and Sabikhi 2020). Measuring the antioxidant power of cheese samples (Figure 2A) showed that the percentage of ABTS inhibition activity, gradually increased with increasing goat's milk content in the cheeses. The highest value of antioxidant capacity was found in sample E after 90 days of ripening (61.26%). The reason could be related to the type of milk in terms of the structure of protein‐forming amino acids and the proteolytic power of the enzymes present in the natural microflora of the milk. The lower intensity of primary and secondary proteolysis in sample E (Figure 1) compared to the other samples also shows the influence of the milk type on peptide formation. Also, the antioxidant power of the samples increased with progress of ripening. Similarly, a gradual increase in the antioxidant capacity of different cheeses during ripening have been reported in the literature (Kocak et al. 2020; Ayyash et al. 2021). The hydrolysis of caseins by microbial proteinases, plasmin, and coagulants during the ripening leads to the formation of free amino acids and water‐soluble peptides with antioxidant properties (Soltani, Guzeler, and Hayaloglu 2015).

### 3.5 α‐Amylase and α‐Glucosidase Activity

Inhibiting the activity of α‐amylase and α‐glucosidase and reducing the rate of carbohydrate hydrolysis are of the effective approaches for controlling the rise in blood glucose and diabetes (Tundis, Loizzo, and Menichini 2010). The inhibitory power of cheese samples against α‐amylase and α‐glucosidase at day 1 and 90 of ripening is shown in Figure 2B,C, respectively. As shown in Figure 2B, α‐amylase inhibitory activity increased after 90 days of ripening in all samples, which could be attributed to the proteolysis of cheese proteins and the release of bioactive peptides during the ripening period (Ayyash et al. 2018). An increase in goat's milk proportion to 75% and 100% (samples D and E) led to a decrease in α‐amylase inhibitory activity.

One of the effective factors in elevation of blood glucose is the activity of α‐glucosidase (EC 3.2.1.20). This enzyme acts on complex carbohydrates and releases the monosaccharides through cleaving of glycosidic bonds (Konrad et al. 2014). In this study, α‐glucosidase inhibitory activity increased with increasing levels of goat's milk in samples at day 90 of ripening (Figure 2C). The highest α‐glucosidase inhibitory activity was observed in sample E after 90 days.

It seems that the presence of peptides with α‐glucosidase inhibitory activity in cheese is related to the amino acid composition of milk, the microbiota of the raw milk, and the proteolytic power of the microorganisms involved in the fermentation process. On the other hand, it has been shown that the degree of hydrophobicity of peptides and their molecular weight can influence their inhibitory activity against the α‐glucosidase (Ren et al. 2016). Peptides with molecular weights less than 2 kDa can inhibit the activity of α‐glucosidase (Ibrahim, Koorbanally, and Islam 2014). Presence of hydrophobic amino acids, especially Leu and Pro, in the structure of peptides greatly inhibit the α‐glucosidase (Ren et al. 2016). The increase of total free hydrophobic amino acids content (Val, Met, Gly, Pro, Ala, Leu, Ileu, Trp, and Phe) in the samples after 90 days of ripening in comparison to these values on the first day of production are shown in Table 1. The results indicate that the release rate of free hydrophobic amino acids increased during 90 days with the elevation of goat's milk proportion, so that samples E and A showed the highest and the lowest increase in the concentration of hydrophobic amino acids on the 90^th^ day of ripening (Table 1). The highest and the lowest inhibitory activities against α‐glucosidase was also observed in samples E and A (after 90 days), respectively (Figure 2C). This result is in agreement with the study performed by Ren et al. (2016) on the increase in α‐glucosidase inhibitory power of peptides composed of hydrophobic amino acids.

It seems that occupation of the active sites of α‐glucosidase by hydrophobic peptides (Zhao et al. 2022) as well as free hydrophobic amino acids, and preventing enzyme‐substrate complex formation is one of the proposed mechanisms for α‐glucosidase inhibition. On the other hand, steric hindrance and electrostatic interactions also play key roles in inhibiting the activity of this enzyme. In the case of C=O, N–H, and other hydrophilic groups, electrostatic interactions play an inhibitory role by occupying the active site of the enzyme, while in the case of some hydrophobic amino acids such as Leu, isopropylene groups inhibit enzyme activity by steric hindrance mechanism (Figure 2D).

### 3.6 FFA Analysis

The FFA content of cheeses increased during 90‐days of ripening (Table 2), indicating the important role of ripening time in the FA profile of the cheeses. The highest concentration of saturated and unsaturated FAs was found in sample (A) after 90 days, which was related to palmitic (C16) and linoleic acid (cis‐9.12 C18:2), respectively. Sample A also contained the highest concentration of elaidic acid as an important trans‐FA. As the proportion of sheep's milk was reduced, the concentration of FAs decreased. The reason is the higher fat content of this milk (6.64%) compared to goat's milk (3.97%) (see section 2: Raw milk specifications). The increase in FA concentration by progress of ripening is related to lipolysis during ripening. FFAs are released by the lipolysis of milk fat. Lipolysis is catalyzed by milk lipoprotein lipase, starter and NSLAB, adjunct cultures or exogenous lipolytic enzymes (Tekin and Güler 2019).

**TABLE 2:** Free fatty acid (mg kg^−1^) analysis of Lighvan cheese samples manufactured with different ratios of sheep to goat's milk at first and 90^th^ day of ripening.

A1	A90	B1	B90	C1	C90	D1	D90	E1	E90
SFA									
C4 15.07 ± 1.01	27.75 ± 1.70	15.25 ± 1.20	19.55 ± 1.70	5.96 ± 0.40	9.70 ± 0.35	4.80 ± 0.50	8.94 ± 0.70	4.26 ± 0.85	7.55 ± 0.55
C6 15.94 ± 1.90	58.54 ± 3.07	15.81 ± 1.30	18.84 ± 1.80	9.76 ± 0.58	11.70 ± 1.80	6.40 ± 0.12	5.98 ± 0.17	7.45 ± 0.98	5.30 ± 0.11
C8 21.96 ± 2.10	134.25 ± 11.51	25.36 ± 2.21	55.15 ± 1.55	13.05 ± 1.02	20.82 ± 1.92	8.69 ± 0.71	10.81 ± 0.99	9.32 ± 1.03	12.78 ± 1.52
C10 98.30 ± 2.42	415.46 ± 20.05	123.54 ± 10.64	223.04 ± 13.95	67.30 ± 1.89	122.70 ± 9.98	37.49 ± 1.90	86.89 ± 2.31	56.82 ± 93	87.59 ± 2.72
C12 75.90 ± 2.18	247.66 ± 13.75	78.35 ± 1.91	147.41 ± 10.61	44.24 ± 1.93	80.04 ± 2.03	26.36 ± 1.75	66.28 ± 2.20	28.99 ± 1.03	42.27 ± 1.77
C14 232.65 ± 15.97	614.47 ± 30.05	239.35 ± 12.32	391.07 ± 12.80	122.77 ± 10.37	224.49 ± 11.06	74.79 ± 2.98	194.29 ± 11.46	70.95 ± 8.87	132.31 ± 12.95
C15 23.67 ± 1.19	71.97 ± 2.31	26.44 ± 1.05	45.94 ± 1.96	14.84 ± 0.93	22.88 ± 1.11	8.84 ± 0.36	16.64 ± 1.09	9.00 ± 0.96	11.13 ± 1.19
C16 871.37 ± 44.80	1972.89 ± 101.19	871.21 ± 43.30	1125.39 ± 99.79	509.98 ± 30.61	688.34 ± 32.05	331.79 ± 20.90	581.62 ± 29.28	370.89 ± 22.32	504.37 ± 28.92
C17 4.08 ± 0.20	16.16 ± 1.11	5.34 ± 0.21	11.44 ± 1.23	4.11 ± 0.19	5.71 ± 0.33	N.D.	N.D.	3.54 ± 0.12	2.81 ± 0.18
C18 311.07 ± 12.51	522.49 ± 26.87	317.44 ± 11.9	363.96 ± 13.05	203.88 ± 11.19	233.07 ± 11.32	145.34 ± 10.81	182.61 ± 10.59	105.68 ± 9.79	101.86 ± 9.92
UFA									
cis‐9 C10:1 2.25 ± 0.05	13.69 ± 0.80	2.27 ± 0.54	4.60 ± 0.65	N.D.	N.D.	N.D.	1.87 ± 0.23	N.D.	1.25 ± 0.10
cis‐9 C14:1 2.33 ± 0.11	6.64 ± 0.69	3.75 ± 0.14	6.83 ± 0.51	2.51 ± 0.09	5.46 ± 0.33	N.D.	4.46 ± 0.66	N.D.	2.14 ± 0.13
cis‐9 C16:1 14.84 ± 1.04	70.13 ± 2.21	15.02 ± 1.67	53.83 ± 2.11	18.61 ± 1.13	18.77 ± 1.10	11.93 ± 0.95	14.61 ± 1.12	5.19 ± 0.87	9.17 ± 0.88
cis‐9 C18:1 22.20 ± 1.80	51.56 ± 1.14	7.99 ± 0.95	37.45 ± 2.15	14.44 ± 1.21	20.18 ± 2.32	7.69 ± 0.92	11.92 ± 0.99	7.86 ± 0.34	11.07 ± 0.88
trans‐9 C18:1616.46 ± 15.46	1306.45 ± 102.49	566.72 ± 11.32	1057.98 ± 99.11	384.11 ± 10.35	558.10 ± 12.01	242.89 ± 9.94	386.95 ± 10.19	341.18 ± 9.88	457.75 ± 10.79
cis‐9.12 C18:2 73.99 ± 3.11	1085.15 ± 97.48	42.17 ± 1.81	120.23 ± 10.24	39.30 ± 1.22	67.04 ± 2.61	21.72 ± 1.25	41.04 ± 1.35	38.44 ± 1.72	38.46 ± 1.39
cis‐9.12.15 C18:3 44.44 ± 2.15	128.10 ± 9.84	36.61 ± 1.06	71.22 ± 3.25	16.98 ± 0.92	31.20 ± 1.10	14.38 ± 0.88	27.12 ± 1.58	5.51 ± 0.39	9.99 ± 0.56

*Note:* A: 100% Sheep's milk + 0% Goat's milk, B: 75% Sheep's milk + 25% Goat's milk, C: 50% Sheep's milk + 50% Goat's milk, D: 25% Sheep's milk + 75% Goat's milk, E: 0% Sheep's milk + 100% Goat's milk.

Abbreviations: SFA, saturated fatty acids; UFA, saturated fatty acids.

### 3.7 Volatile Compounds

**FIGURE 3:**
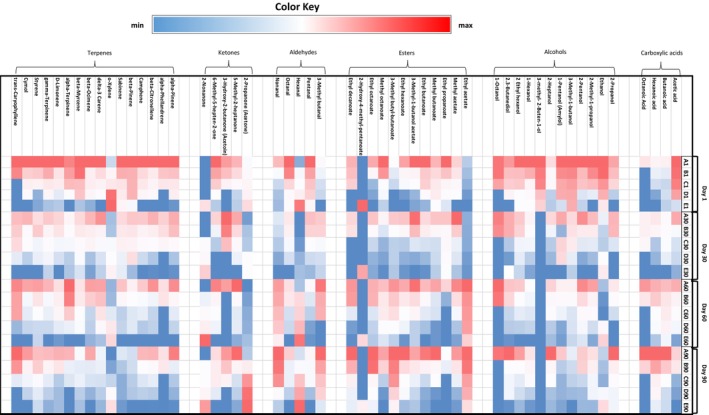
Variation of the main chemical groups of volatile compounds of Lighvan cheeses manufactured with different ratios of sheep and goat's milk during the ripening period. Color ranging from blue to red corresponds to minimum to maximum levels of each compound in a row. A: 100% Sheep's milk + 0% Goat's milk, B: 75% Sheep's milk + 25% Goat's milk, C: 50% Sheep's milk + 50% Goat's milk, D: 25% Sheep's milk + 75% Goat's milk, E: 0% Sheep's milk + 100% Goat's milk.

**FIGURE 4:**
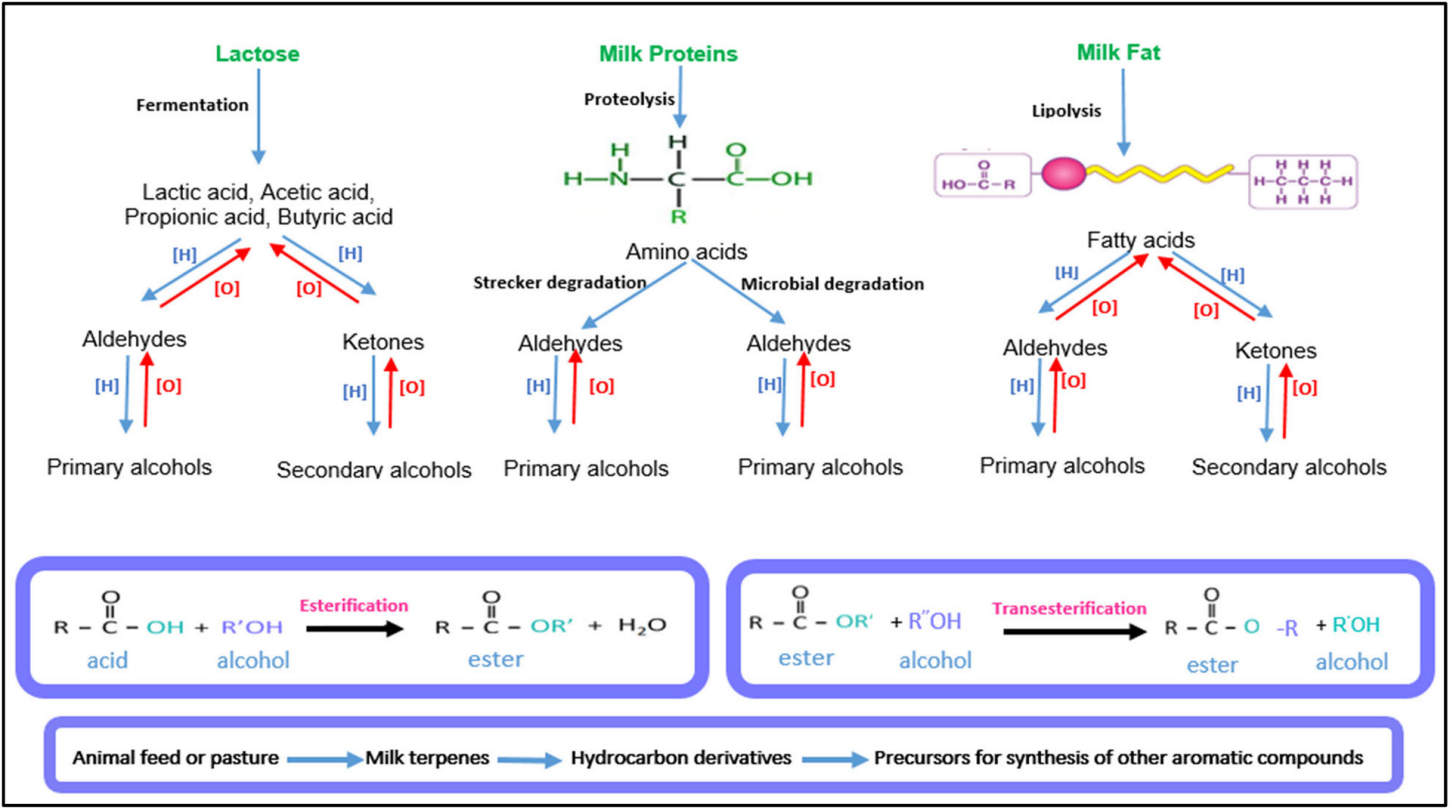
The mechanisms for volatile compounds generation in a cheese.

Volatile compounds are formed through complex chemical, microbial, and enzymatic reactions during the making and ripening of a cheese and significantly influence the final taste of this product. The characteristics of the milk (the content of protein, fat, saturated and unsaturated fatty acids, and the type of amino acids), animal feed quality, ripening duration and the microbial flora involved in the cheese‐making process play important roles in the formation of these compounds. In the present study, seven groups of chemical compounds were detected in the Lighvan cheese samples, including acids, aldehydes, alcohols, esters, ketones, terpenes, and miscellaneous compounds. Figures 3 and 4 show the changes in volatiles in different cheese samples during 90‐days of ripening and the proposed mechanisms for formation of volatile compounds in a cheese, respectively.

#### 3.7.1 Carboxylic Acids

The presence of carboxylic acids in a cheese is the result of the fermentation and ripening phenomenon. Acetic acid is produced by the metabolism of lactose, amino acids or citrate and is responsible for the “pungent” flavor note in the cheese (Ozturkoglu‐Budak et al. 2016; De Jesus et al. 2021). This compound decreased in the cheese samples after 90 days. This decrease was consistent with a notable increase in ethyl acetate (Figure 3). Tekin and Guler (2021) reported a similar result in Tulum cheese during its ripening. Fatty acids also release by lipolysis during ripening and affect the flavor of the cheese (Figure 4). An increase in C4–C8 concentration was observed in all Lighvan samples after 90 days of ripening (Figure 3). In addition, the concentration of organic acids decreased with increasing proportion of goat's milk during ripening. This may be due to the lower amount of lactose and fat in goat's milk compared to sheep's milk (Park et al. 2007).

#### 3.7.2 Alcohols

The reduction of aldehydes and ketones or the transesterification reaction (Tekin and Guler 2021) leads to the formation of primary or secondary alcohols (Figure 4). As can be seen in Figure 3, the ratio of sheep's to goat's milk significantly influenced the alcohol content (*p* < 0.05). On all days tested, the samples with higher proportions of sheep's milk contained more alcohol. As ripening progressed, the concentration of alcohols decreased (*p* < 0.05). Some alcohols, such as 3‐methyl‐2‐buten‐1‐ol and 1‐octanol, showed higher concentrations from day 30 onwards. Ethanol, 2‐methyl‐1‐propanol, 2‐pentanol, 1‐pentanol, 3‐methyl‐1‐butanol, 3‐methyl‐2‐butane‐1‐ol, 1‐hexanol, 2‐ethylhexanol, and 1‐octanol were alcohols that appeared in higher concentrations in sample A, especially in the fresh cheese. Ethanol formation is the result of lactose fermentation and amino acid metabolism. Although ethanol has little effect on cheese flavor, it favors the formation of ethyl esters (Atik et al. 2021). In the current study, a decrease in ethanol after 90 days and an increase in the concentration of ethyl esters (especially ethyl acetate) was observed. 3‐Methyl‐1‐butanol is responsible for the development of alcoholic and green notes in a cheese. High levels of 3‐methyl‐1‐butanol have been reported in other brined cheeses such as Macedonian, Domiati and Gokceada (Tekin and Hayaloglu 2023). This compound can be synthesized from Leu or synthesized by the reduction of 3‐methyl‐1‐butanal (Oluk 2023).

#### 3.7.3 Esters

Esters are generated by the esterification reaction between acids and alcohols. Alos, transesterification took place between an ester and an alcohol, and lead to the formation of new esters (Figure 4). These compounds have a low perception threshold and give a cheese a fruity, sweet and floral taste. They can delay the sentience of undesirable aromas caused by some carboxylic acids, aldehydes, and amines (De Jesus et al. 2021). In general, the proportion of milk and ripening duration significantly affected the concentration of esters (*p* < 0.05). The samples with a higher proportion of sheep's milk had higher amounts of ester compounds (Figure 3). Ethyl acetate increased significantly in all samples from the 60th day of ripening. The reaction of ethanol and acetic acid leads to the formation of ethyl acetate. A decrease in the concentration of ethanol and acetic acid after 30 days of ripening in all samples are clearly observed in Figure 3. The presence of ethyl acetate has been documented in Tulum (Atik et al. 2021) and Turkish white cheeses (Oluk 2023). Ethyl butanoate, ethyl propanoate, ethyl hexanoate, ethyl decanoate, and ethyl octanoate, as well as 3‐methyl butyl butanoate, were other ethyl esters detected in the cheeses. Ethyl hexanoate, ethyl propanoate, ethyl decanoate and ethyl octanoate had higher concentrations in A and B samples. A decrease in the concentration of ethyl propanoate and a slight increase in the concentration of the other mentioned esters was observed with progress of ripening. The presence of ethyl butanoate, ethyl octanoate and ethyl hexanoate was detected in Turkish Divle Cave cheese made from raw sheep's milk (Ozturkoglu‐Budak et al. 2016).

#### 3.7.4 Aldehydes

Five types of aldehydes (3‐methylbutanal, pentanal, hexanal, octanal, and nonanal) were detected in the current study (Figure 3). An increase in the proportion of goat's milk led to a decrease in the concentration of 3‐methylbutanal, pentanal, octanal and nonanal but increased the concentration of hexanal. 3‐Methylbutanal is a branched‐chain aldehyde. It is produced from Leu and is characterized by a green, malty odor (pungent) that becomes fruity and almond‐like at low concentrations (De Jesus et al. 2021; Oluk 2023). The highest amount of this compound was detected in sample A after 90 days. Straight‐chain aldehydes such as pentanal, hexanal, octanal and nonanal exhibit aromas of green grass and herbs (Ozturkoglu‐Budak et al. 2016). The concentration of pentanal and octanal decreased by progress of ripening. Hexanal concentration decreased in all samples during the first 30 days of ripening but a slight increase was observed during the remaining maturation period.

#### 3.7.5 Ketones

Ketones generate fruity, floral, and herbal notes in a cheese (Bezerra et al. 2017). The reduction of carboxylic acids, oxidation of alcohols, or the oxidation of fatty acids to β‐keto acids and their further decarboxylation lead to the formation of aromatic ketones (Suzuki‐Iwashima et al. 2020) (Figure 4). Five types of ketones (2‐propanone, 5‐methyl‐2‐heptanone, 3‐hydroxy‐2‐butanone (acetoin), 2‐nonanone, and 6‐methyl‐5‐hepten‐2‐one) were identified in cheese samples during their ripening (Figure 3). Significant differences were determined in the concentration of the identified ketones when the ratio of sheep's to goat's milk and the ripening time were changed (*p* < 0.05). Among the detected ketones, the concentration of 2‐propanone and 2‐nonanone increased with the increase in the proportion of goat's milk. After 90 days, sample E contained the highest content of the mentioned compounds (Figure 3). Kondyli, Pappa, and Svarnas (2016) reported the high content of 2‐propanone in white goat cheese. The increase in the concentration of 2‐nonanone as an effective factor in producing floral, fruity and peachy notes in Tulum cheese (made from raw goat milk) up to 180 days of ripening has been documented (Atik et al. 2021). The concentrations of 5‐methyl‐2‐heptanone, acetoin, and 6‐methyl‐5‐hepten‐2‐one decreased with increasing in proportion of goat's milk. 5‐methyl‐2‐heptanone showed no significant changes during ripening (Figure 3). The concentration of acetoin, a compound responsible for fresh cheese, fat and butter flavors (Tekin and Guler 2021), fluctuated during 90 d of ripening.

#### 3.7.6 Terpenes

The presence of terpenes in cheese is associated with animal feed or pasture (Tekin and Guler 2021). Different types of terpenes including α‐pinene, β‐pinene, β‐citronellene, camphene, sabinene, o‐xylene, δ‐3‐carene, β‐ocimene, β‐myrcene, α‐terpinene, D‐limonene, γ‐terpinene, styrene, cymene, α‐phellandrene, and trans‐caryophyllene were identified in the cheese samples (Figure 3). In general, the samples made with higher proportions of sheep's milk contained higher percentage of terpenes, with the exception of O‐xylene, which appeared in higher concentrations in the fresh cheeses made with high percentages of goat's milk (Figure 3). The presence of O‐xylene in Tulum cheese manufactured from raw goat's milk has been reported by Atik et al. (2021). The ripening time also significantly influenced the concentration of terpenes (*p* < 0.05). Almost the amount of all measured terpenes decreased with progress of ripening.

## 4 Conclusion

This study investigated the influence of various blends of sheep's and goat's milk on the functional metabolites and volatile compounds produced in Lighvan cheese throughout ripening. The results showed significant influence of mixing ratio and ripening time on the synthesis of functional metabolites. Increasing the ratio of sheep's to goat's milk and extending the ripening time led to a higher content of TFAA. A slightly higher GABA content was found in cheeses with a higher proportion of sheep's milk. Increase in the proportion of goat's milk led to a significant increase in the antioxidant activity and inhibitory power of the samples against ACE and α‐glucosidase. Various volatile compounds were detected in the analyzed samples during their ripening in brine. The results of this study show that the production of functional Lighvan cheese is possible by intelligently selecting the type of milks used and monitoring the ripening time of the cheese in the brine. These results are important for the industrial production of functional Lighvan cheese with antihypertensive, antioxidant, or antidiabetic properties, while maintaining the authenticity of traditional raw milk cheeses. The use of certain types of starters and NSLAB is proposed to achieve higher concentrations of the desired bioactive compounds.

## Author Contributions

**Ali Tekin:** formal analysis (equal), methodology (equal), software (equal), validation (equal). **Hilal Kanmaz:** formal analysis (equal), methodology (equal), software (equal), validation (equal). **Didem Sahingil:** formal analysis (equal), methodology (equal), software (equal), validation (equal). **Büşra Kaya:** formal analysis (equal), methodology (equal), software (equal), validation (equal). **Yasemin Gökçe:** formal analysis (equal), methodology (equal), software (equal), validation (equal). **Ali Adnan Hayaloglu:** funding acquisition (equal), supervision (equal), visualization (equal), writing – review and editing (supporting). **Solmaz Saremnezhad:** conceptualization, project administration, funding acquisition, investigation, writing ‐ original draft. **Mostafa Soltani:** data curation, supervision, funding acquisition.

## Conflicts of Interest

The authors declare no conflicts of interest.

## Data Availability

The data that support the findings of this study are available from the corresponding author upon reasonable request.

## References

[fsn34543-bib-0001] Atik, D. S. , N. Akın , H. C. Akal , and C. Koçak . 2021. “The Determination of Volatile Profile During the Ripening Period of Traditional Tulum Cheese From Turkey, Produced in Anamur in the Central Taurus Region and Ripened in Goatskin.” International Dairy Journal 117: 104991.

[fsn34543-bib-0002] Ayyash, M. , A. Abdalla , M. Alameri , et al. 2021. “Biological Activities of the Bio Accessible Compounds After In Vitro Digestion of Low‐Fat Akawi Cheese Made From Blends of Bovine and Camel Milk.” Journal of Dairy Science 104, no. 9: 9450–9464. 34147215 10.3168/jds.2021-20438

[fsn34543-bib-0003] Ayyash, M. , A. K. Al‐Nuaimi , S. Al‐Mahadin , and S. Q. Liu . 2018. “In Vitro Investigation of Anticancer and ACE‐Inhibiting Activity, α‐Amylase and α‐Glucosidase Inhibition, and Antioxidant Activity of Camel Milk Fermented With Camel Milk Probiotic: A Comparative Study With Fermented Bovine Milk.” Food Chemistry 239: 588–597. 28873609 10.1016/j.foodchem.2017.06.149

[fsn34543-bib-0004] Bezerra, T. K. A. , A. N. M. de Oliveira , A. R. R. de Araújo , et al. 2017. “Volatile Profile in Goat Coalho Cheese Supplemented With Probiotic Lactic Acid Bacteria.” LWT–Food Science and Technology 76: 209–215.

[fsn34543-bib-0005] Cam, M. , B. Basyigit , H. Alasalvar , et al. 2020. “Bioactive Properties of Powdered Peppermint and Spearmint Extracts: Inhibition of Key Enzymes Linked to Hypertension and Type 2 Diabetes.” Food Bioscience 35: 100577.

[fsn34543-bib-0006] Campagnollo, F. B. , G. T. Pedrosa , B. A. Kamimura , et al. 2022. “Growth Potential of Three Strains of Listeria Monocytogenes and Salmonella Enterica in Frescal and Semi‐Hard Artisanal Minas Microcheeses: Impact of the Addition of Lactic Acid Bacteria With Antimicrobial Activity.” LWT–Food Science and Technology 158: 113169.

[fsn34543-bib-0007] Da Silva, D. D. , M. D. S. F. de Lima , M. F. da Silva , et al. 2019. “Bioactive Water‐Soluble Peptides From Fresh Buffalo Cheese May Be Used as Product Markers.” LWT–Food Science and Technology 108: 97–105.

[fsn34543-bib-0008] De Jesus, F. M. , B. Klein , R. Wagner , and H. T. Godoy . 2021. “Key Aroma Compounds of Canastra Cheese: HS‐SPME Optimization Assisted by Olfactometry and Chemometrics.” Food Research International 150: 110788. 34865803 10.1016/j.foodres.2021.110788

[fsn34543-bib-0009] Dhakal, R. , V. K. Bajpai , and K. H. Baek . 2012. “Production of GABA (γ‐Aminobutyric Acid) by Microorganisms: A Review.” Brazilian Journal of Microbiology 43: 1230–1241. 24031948 10.1590/S1517-83822012000400001 PMC3769009

[fsn34543-bib-0010] Fox, P. F. , and P. L. McSweeney . 2017. “Cheese: An Overview.” In Cheese: Chemistry, Physics and Microbiology, edited by P. F. Fox , P. L. H. McSweeney , P. D. Cotter , and D. W. Everett , 5–21. London, UK: Elsevier Academic Press.

[fsn34543-bib-0011] Hayaloglu, A. A. 2017. “Cheese Varieties Ripened Under Brine.” In Cheese: Chemistry, Physics and Microbiology, edited by P. L. H. McSweeney , P. F. Fox , P. D. Cotter , and D. W. Everett , 4th ed. 997–1040. London, UK: Academic Press.

[fsn34543-bib-0012] Hayaloglu, A. A. , M. Guven , P. F. Fox , and P. L. H. Mc Sweeney . 2005. “Influence of Starters on Chemical, Biochemical, and Sensory Changes in Turkish White‐Brined Cheese During Ripening.” Journal of Dairy Science 88, no. 10: 3460–3474. 16162519 10.3168/jds.S0022-0302(05)73030-7

[fsn34543-bib-0013] Hossain, S. , Y. Khetra , S. Ganguly , R. Kumar , and L. Sabikhi . 2020. “Effect of Heat Treatment on Plasmin Activity and Bio‐Functional Attributes of Cheddar Cheese.” LWT–Food Science and Technology 120: 108924.

[fsn34543-bib-0014] IBM Corp . 2013. IBM SPSS Statistics for Windows, Version 22.0. Armonk, NY: IBM Corp.

[fsn34543-bib-0015] Ibrahim, M. A. , N. A. Koorbanally , and M. S. Islam . 2014. “Anti‐Oxidative Activity and Inhibition of Key Enzymes Linked to Type 2 Diabetes (α‐Glucosidase and α‐Amylase) by *Khaya Senegalensis* .” Acta Pharmaceutica 64, no. 3: 311–324. 25296677 10.2478/acph-2014-0025

[fsn34543-bib-0016] İncili, G. K. , M. Akgöl , P. Karatepe , et al. 2023. “Whole‐Cell Postbiotics: An Innovative Approach for Extending the Shelf Life and Controlling Major Foodborne Pathogens in Chicken Breast Fillets.” Food and Bioprocess Technology 16, no. 7: 1–23.

[fsn34543-bib-0017] Kocak, A. , T. Sanli , E. A. Anli , and A. A. Hayaloglu . 2020. “Role of Using Adjunct Cultures in Release of Bioactive Peptides in White‐Brined Goat‐Milk Cheese.” LWT–Food Science and Technology 123: 109127.

[fsn34543-bib-0018] Kondyli, E. , E. C. Pappa , and C. Svarnas . 2016. “Ripening Changes of the Chemical Composition, Proteolysis, Volatile Fraction and Organoleptic Characteristics of a White‐Brined Goat Milk Cheese.” Small Ruminant Research 145: 1–6.

[fsn34543-bib-0019] Konrad, B. , D. Anna , S. Marek , P. Marta , Z. Aleksandra , and C. Józefa . 2014. “The Evaluation of Dipeptidyl Peptidase (DPP)‐IV. α‐Glucosidase and Angiotensin Converting Enzyme (ACE) Inhibitory Activities of Whey Proteins Hydrolyzed With Serine Protease Isolated From Asian Pumpkin (*Cucurbita ficifolia*).” International Journal of Peptide Research and Therapeutics 20, no. 4: 483–491. 25364320 10.1007/s10989-014-9413-0 PMC4210635

[fsn34543-bib-0020] Miloradovic, Z. , N. Kljajevic , J. Miocinovic , N. Tomic , J. Smiljanic , and O. Macej . 2017. “High Heat Treatment of Goat Cheese Milk. The Effect on Yield, Composition, Proteolysis, Texture and Sensory Quality of Cheese During Ripening.” International Dairy Journal 68: 1–8.

[fsn34543-bib-0021] Oluk, A. C. 2023. “Effect of Production Variations on the Composition, Textural and Microstructural Properties, and Volatile Compounds of Turkish White Cheese During Ripening.” LWT–Food Science and Technology 173: 114348.

[fsn34543-bib-0022] Ozturkoglu‐Budak, S. , A. Gursoy , D. P. Aykas , et al. 2016. “Volatile Compound Profiling of Turkish Divle Cave Cheese During Production and Ripening.” Journal of Dairy Science 99, no. 7: 5120–5131. 27108178 10.3168/jds.2015-10828

[fsn34543-bib-0023] Park, Y. W. , M. Juárez , M. Ramos , and G. F. W. Haenlein . 2007. “Physico‐Chemical Characteristics of Goat and Sheep Milk.” Small Ruminant Research 68, no. 1–2: 88–113.

[fsn34543-bib-0024] Ramos, L. Á. , D. A. Baez , G. D. Ortiz , J. C. R. Ruiz , and V. M. T. López . 2022. “Antioxidant and Antihypertensive Activity of Gouda Cheese at Different Stages of Ripening.” Food Chemistry: X 14: 100284. 35345793 10.1016/j.fochx.2022.100284 PMC8956798

[fsn34543-bib-0025] Redruello, B. , A. Szwengiel , V. Ladero , B. del Rio , and M. A. Alvarez . 2020. “Identification of Technological/Metabolic/Environmental Profiles of Cheeses With High GABA Contents.” LWT–Food Science and Technology 130: 109603.

[fsn34543-bib-0026] Ren, Y. , K. Liang , Y. Jin , et al. 2016. “Identification and Characterization of Two Novel α‐Glucosidase Inhibitory Oligopeptides From Hemp (*Cannabis sativa L*.) Seed Protein.” Journal of Functional Foods 26: 439–450.

[fsn34543-bib-0027] Sahingil, D. , Y. Gokce , M. Yuceer , and A. A. Hayaloglu . 2019. “Optimization of Proteolysis and Angiotensin Converting Enzyme Inhibition Activity in a Model Cheese Using Response Surface Methodology.” LWT–Food Science and Technology 99: 525–532.

[fsn34543-bib-0028] Saidi, V. , M. Sheikh‐Zeinoddin , F. Kobarfard , S. Soleimanian‐Zad , and A. S. Doost . 2020. “Profiling of Bioactive Metabolites During the Ripening of a Semi‐Hard Non‐Starter Culture Cheese to Detect Functional Dietary Neurotransmitters.” Biocatalysis and Agricultural Biotechnology 28: 101734.

[fsn34543-bib-0029] Saremnezhad, S. , M. Soltani , A. Faraji , and A. A. Hayaloglu . 2021. “Chemical Changes of Food Constituents During Cold Plasma Processing: A Review.” Food Research International 147: 110552. 34399529 10.1016/j.foodres.2021.110552

[fsn34543-bib-0030] Soltani, M. , N. Guzeler , and A. A. Hayaloglu . 2015. “The Influence of Salt Concentration on the Chemical, Ripening and Sensory Characteristics of Iranian White Cheese Manufactured by UF‐Treated Milk.” Journal of Dairy Research 82, no. 3: 365–374. 26119429 10.1017/S0022029915000278

[fsn34543-bib-0031] Soltani, M. , S. Saremnezhad , A. R. Faraji , and A. A. Hayaloglu . 2022. “Perspectives and Recent Innovations on White Cheese Produced by Conventional Methods or Ultrafiltration Technique.” International Dairy Journal 125: 105232.

[fsn34543-bib-0032] Soodam, K. , and T. P. Guinee . 2018. “The Case for Milk Protein Standardization Using Membrane Filtration for Improving Cheese Consistency and Quality.” International Journal of Dairy Technology 71, no. 2: 277–291.

[fsn34543-bib-0033] Sulejmani, E. , D. Sahingil , and A. A. Hayaloglu . 2020. “A Comparative Study of Compositional, Antioxidant Capacity, ACE‐Inhibition Activity, RP‐HPLC Peptide Profile and Volatile Compounds of Herbal Artisanal Cheeses.” International Dairy Journal 111: 104837.

[fsn34543-bib-0034] Suzuki‐Iwashima, A. , H. Matsuura , A. Iwasawa , and M. Shiota . 2020. “Metabolomics Analyses of the Combined Effects of Lactic Acid Bacteria and *Penicillium camemberti* on the Generation of Volatile Compounds in Model Mold‐Surface‐Ripened Cheese.” Journal of Bioscience and Bioengineering 129, no. 3: 333–347. 31611057 10.1016/j.jbiosc.2019.09.005

[fsn34543-bib-0035] Tekin, A. , and Z. Güler . 2019. “Glycolysis, Lipolysis and Proteolysis in Raw Sheep Milk Tulum Cheese During Production and Ripening: Effect of Ripening Materials.” Food Chemistry 286: 160–169. 30827590 10.1016/j.foodchem.2019.01.190

[fsn34543-bib-0036] Tekin, A. , and Z. Guler . 2021. “The Effect of Ripening Medium (Goat Skin Bag or Plastic Barrel) on the Volatile Profile, Color Parameter and Sensory Characteristics of Tulum Cheese.” Journal of Central European Agriculture 22, no. 1: 19–38.

[fsn34543-bib-0037] Tekin, A. , and A. A. Hayaloglu . 2023. “Understanding the Mechanism of Ripening Biochemistry and Flavor Development in Brine Ripened Cheeses.” International Dairy Journal 137: 105508.

[fsn34543-bib-0038] Tundis, R. , M. R. Loizzo , and F. Menichini . 2010. “Natural Products as α‐Amylase and α‐Glucosidase Inhibitors and Their Hypoglycaemic Potential in the Treatment of Diabetes: An Update.” Mini Reviews in Medicinal Chemistry 10, no. 4: 315–331. 20470247 10.2174/138955710791331007

[fsn34543-bib-0039] Uruc, K. , A. Tekin , D. Sahingil , and A. A. Hayaloglu . 2022. “An Alternative Plant‐Based Fermented Milk With Kefir Culture Using Apricot (*Prunus armeniaca L*.) Seed Extract: Changes in Texture, Volatiles and Bioactivity During Ewe.” Innovative Food Science & Emerging Technologies 82: 103189.

[fsn34543-bib-0040] Wang, S. , L. Zhang , H. Wang , J. Liu , Y. Hu , and Z. Tu . 2024. “Angiotensin Converting Enzyme (ACE) Inhibitory Peptide From the Tuna (*Thunnus thynnus*) Muscle: Screening, Interaction Mechanism and Stability.” International Journal of Biological Macromolecules 279: 135469. 39250996 10.1016/j.ijbiomac.2024.135469

[fsn34543-bib-0041] Zhao, Q. , G. Wei , K. Li , S. Duan , R. Ye , and A. Huang . 2022. “Identification and Molecular Docking of Novel α‐Glucosidase Inhibitory Peptides From Hydrolysates of Binglangjiang Buffalo Casein.” LWTFood Science and Technology 156: 113062.

